# Finding carcinoid tumor before bariatric surgery. Is preoperative endoscopy necessary? Case report

**DOI:** 10.1016/j.ijscr.2019.08.011

**Published:** 2019-08-20

**Authors:** Ozan Şen, Ahmet Gökhan Türkçapar

**Affiliations:** a Türkçapar Bariatrics, Obesity Center, Istanbul, Turkey; b Acibadem Fulya Hospital, Istanbul, Turkey

**Keywords:** Bariatric surgery, Preoperative endoscopy, Neuroendocrine tumor

## Abstract

**Introduction:**

Carcinoid tumors are endocrine system-related lesions and 4% of the gastrointestinal tract's neuroendocrine tumors (NET) originate from stomach. In recent years, gastric carcinoid tumors have been reported at increasing rates on endoscopies. In this article, we will present a case of gastric carcinoid tumor detected at the upper gastrointestinal (GI) endoscopy during preoperative bariatric surgery workup.

**Case presentation:**

A 55 years old male patient with body mass index (BMI) 46 kg/m^2^ was scheduled for bariatric surgery. Upper GI endoscopy revealed 2 separate 4–5 mm nodular lesions at gastric corpus and antrum. Biopsies were taken and both lesions were reported as neuroendocrine tumors. It was decided that the Laparoscopic sleeve gastrectomy (LSG) operation would be performed because both lesion areas would remain in the extracted part of stomach.

**Discussion:**

The routine use of upper GI endoscopy in preoperative evaluation of bariatric surgery patients still remains controversial.

**Conclusion:**

Upper GI endoscopy is very important in determining various gastric pathologies and determining the most appropriate surgical method before bariatric surgery.

## Highlights

• The routine use of upper GI endoscopy in preoperative evaluation of bariatric surgery patients still remains controversial.
• The incidence of cancer increases in obese patients.
• The incidence of gastric neuroendocrine tumors in obese patients is higher than the general population (0.6% to 0.0006%).
• Endoscopic evaluation before bariatric surgery is important in terms of revealing many stomach pathologies that may change the treatment of the patient.

## 1 Introduction

Carcinoid tumors frequently occur in the gastrointestinal tract (70%). The tumors are endocrine system-related lesions and 4% of the gastrointestinal tract’s NETs originate from stomach [[1], [2], [3]]. Gastric carcinoids can be divided into 3 subgroups. Type 1 constitues 70–80% of the stomach carcinoids and develops often on the basis of chronic atrophic gastritis and pernicious anemia. This type is associated with achlorhydria and secondary hypergastrinemia caused by parietal cell loss. Type 2 stomach gastric carcinoids occur in 5–10% of cases and are frequently associated with Zollinger Ellison syndrome. Type 1 and type 2 are usually small, multiple and hypergastrinemia related lesions they tend to be mostly confined to the mucosa and submucosa, and rarely metastasize to lymph nodes. Type 3 gastric carcinoids are seen in 15–20% and are usually seen in the elderly. They are more aggressive and unrelated to gastrin level [4]. Treatment depends on the type of tumor. Small localized tumors are suitable for endoscopic polypectomy and mucosal resection [5].

Gastric carcinoid tumors have been increasingly reported in endoscopies performed before bariatric surgery in recent years [6,7]. In this report, we present a case of carcinoid tumor detected by preoperative upper GI endoscopy in a morbidly obese patient prepared for bariatric surgery.

This case report was reported in accordance with the SCARE criteria [16].

## 2 Case report

**Fig. 1:**
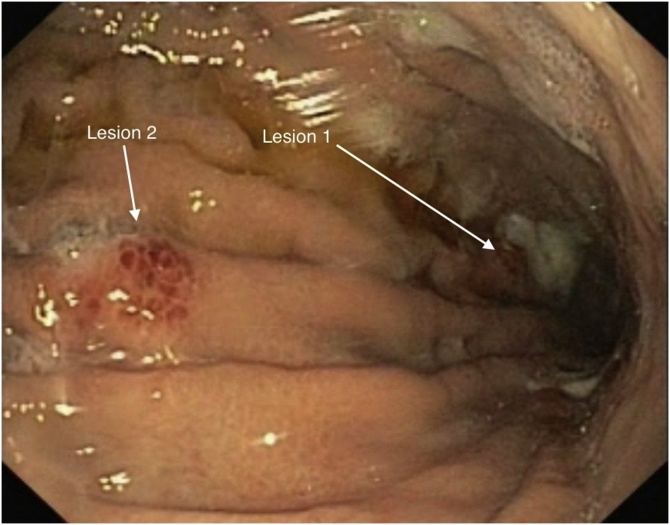
Endoscopic view of lesions.

A 55 years old male patient with BMI 46 kg/m^2^ was scheduled for bariatric surgery. He had type 2 diabetes, hypertension, obstructive sleep apnea and dyslipidemia as comorbid problems. Upper GI endoscopy, which is a part of our preoperative workup, was performed in this patient as in all patients. Endoscopy revealed 2 separate 4–5 mm nodular lesions in gastric corpus and antrum localization (Fig. 1).

**Fig. 2:**
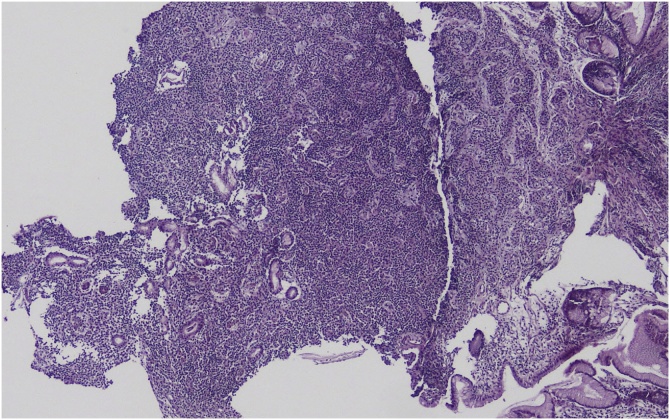
Microscopic view of carcinoid tumor.

Biopsies were taken from two lesions separately. Other stomach areas were in normal appearance. Both lesions were reported as neuroendocrine tumor (Fig. 2). Immunohistochemically; chromogranin A and synaptophysin was reported positive. KI proliferation index was reported as 4%. Both tumors were reported as having smilar characteristics (WHO grade 2). On biopsy specimen taken from stomach corpus, inactive chronic gastritis and incomplete type focal intestinal metaplasia in the environment was detected. The patient’s serum gastrin level was checked and it was detected as high (gastrin level 660). It was decided that Laparoscopic sleeve gastrectomy (LSG) would be performed on the patient because both lesion areas would remain in the extracted part of the stomach.

**Fig. 3:**
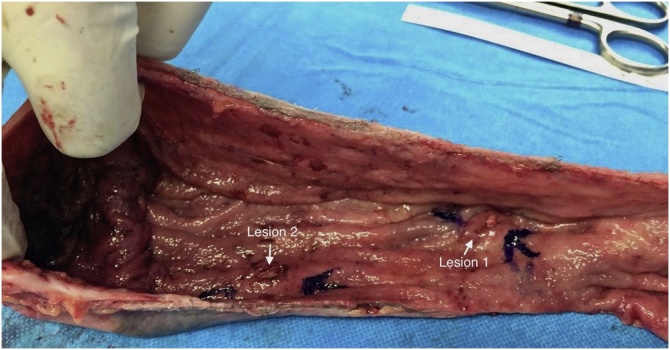
Extracted stomach and lesions.

The patient underwent a sleeve gastrectomy. In addition, intraoperative endoscopy was performed to ensure complete removal of the lesions. When the extracted specimen was opened, both lesions were removed at a distance from the stapler line (Fig. 3). The patient was discharged on the 3rd day with liquid diet in accordance with our routine protocol. There were no complications. Histological examination of the gastric specimen revealed no residual tumor. The rate of excess body weight loss (%EWL) was determined 55% in the 6th month and 71% in the 12th month. Endoscopic follow-up was planned in addition to the controls within the framework of our follow-up program after the operation.

## 3 Discussion

The incidence of cancer increases in obese patients [8]. The incidence of gastric neuroendocrine tumors in obese patients was reported in the literature between 0.6% and 0.8% percent [9,10]. This rate is higher than the general population (0.0006%–0.0015%). Although gastric neuroendocrine tumor is found rarely, the diagnostic frequency has increased with an increase of upper GI endoscopy applications. In this study, we present a neuroendocrine tumor incidentally detected in upper GI endoscopy, as a part of our routine preparation protocol before bariatric surgery.

The routine use of upper GI endoscopy in preoperative evaluation of bariatric surgery patients still remains controversial. The European for Endoscopic Surgery Association recommends endoscopy before bariatric surgery for all patients [11]. In contrast, the American Society of Gastrointestinal and Endoscopic Surgery recommends endoscopy in cases where gastric pathology is suspected [12]. According to a study conducted by the national health unit in England, preoperative endoscopy practice among bariatric centers shows wide variation. Centers are divided into those who routinely perform and selectively perform endoscopy. 10% of these centers find preoperative endoscopy unnecessary [13].

Endoscopic findings, including benign pathologies, have a change in surgical treatment in bariatric surgery patients. When only premalignant and malignant pathologies are considered, this rate decreases to 0.4% [14]. When we look at the bariatric surgical methods used today, sleeve gastrectomy and gastric bypass are the most common methods used worldwide. Inability to reach the remnant stomach is an important problem in patients undergoing gastric bypass. Many surgeons believe that this situation necessitates preoperative gastric endoscopy. As in our case, identification of gastric lesions before bariatric surgery can be extremely important in determining the surgical method of choice. In our patient, LSG was preferred, which allows the resection of two small carcinoid lesions, which were detected incidentally. One reason was the possibility of endoscopic evaluation during follow-up. The development of gastric cancer in the background of intestinal metaplasia is extremely low. This ratio is 2.2% in low-risk populations [15]. As in our patient, the detection of gastric intestinal metaplasia in endoscopic pathologies may change the treatment choice because it provides better follow-up in the future.

## 4 Conclusion

There are many methods in the surgical treatment of morbid obesity. Individual factors are effective in determining the appropriate surgical method. Upper gastrointestinal endoscopy is very important in determining various gastric pathologies and determining the most appropriate surgical method before the bariatric surgery

## Funding

No funding.

## Ethical approval

The patient have given her informed consent for this publication. It is exemption from ethical approval because it is an observation report.

## Consent

He gave us his consent.

Written informed consent was obtained from the patient for publication of this case report and accompanying images. A copy of the written consent is available for review by the Editor-in-Chief of this journal on request.

## Author contribution

Each author have participated sufﬁciently in the work to take public responsibility for appropriate portions of the content. All authors met all of the following criteria:

- Substantial contributions to the conception or design of the work; or the acquisition, analysis, or interpretation of data for the work; OS and AT

- Drafting the work or revising it critically for important intellectual content; OS

- Final approval of the version to be published; OS

- Agreement to be accountable for all aspects of the work in ensuring that questions related to the accuracy or integrity of any part of the work are appropriately investigated and resolved.

AT and OS operated the patient.

OS wrote the ﬁrst draft of the manuscript.

OS and AT wrote the ﬁnal draft of the manuscript.

OS made the corrections in English. All authors have reaf and approved the final report.

## Registration of research studies

Not applicable.

## Guarantor

On the behalf of all author I am the guarantor.

Ozan Sen.

## Provenance and peer review

Not commissioned, externally peer-reviewed.

## Declaration of Competing Interest

No conflict of interest.
